# A challenging diagnosis for thiamine transporter deficiency anemia (Rogers syndrome) in two young siblings: A rare case report

**DOI:** 10.1002/ccr3.7192

**Published:** 2023-04-19

**Authors:** Yara Deeb, Nafiza Martini, Yara Ahmad Ahmad, Marwa Alkosti, Othman Hamdan

**Affiliations:** ^1^ Faculty of Medicine Al Andalus University Qadmus Syrian Arab Republic; ^2^ Stemosis for Scientific Research Damascus Syrian Arab Republic; ^3^ Faculty of Medicine Damascus University Damascus Syrian Arab Republic; ^4^ Pediatric University Hospital Damascus Syrian Arab Republic

**Keywords:** case report, diabetes mellitus, megaloblastic anemia, sensorineural deafness, thiamine‐responsive megaloblastic anemia

## Abstract

We present these two cases to emphasize the necessity of critical thinking and high suspicion of the disease (Rogers syndrome) to avoid potentially fatal situations due to its rarity and the importance of early treatment.

## 1 INTRODUCTION

Thiamine‐responsive megaloblastic anemia (TRMA) also known as Rogers syndrome is a rare autosomal recessive disorder in which the majority of cases occur in consanguineous marriages. Thiamine‐responsive megaloblastic anemia is the result of mutation of the *SLC19A2* gene that encodes thiamine transporter protein. Disease onset spans from infancy to adolescence and is characterized by the clinical triad (megaloblastic anemia, sensorineural deafness, and nontype 1 DM), other signs can be seen (including: congenital heart diseases, optic atrophy, retinal dysfunction, and cerebrovascular accidents). It is managed with a lifelong pharmacological dose of thiamine (50–100 mg/day).^1^, ^2^

Herein, we present the case of two sisters with TRMA, to describe the clinical characteristics of the disease and to stress the importance of a well‐educated differential diagnosis and the importance of eliminating cognitive biases. To the best of our knowledge, this is the first case of TRMA published in Syria.

## 2 CASE PRESENTATION

Herein, we present two cases of two siblings born into a consanguineous marriage. Their parents are third‐degree relatives. The two sisters presented with relatively similar symptoms in a different time frames. The presentations of those two cases show the importance of well‐thought differential diagnosis in avoiding catastrophic outcomes.

## 3 CASE 1

The first case was a five‐month‐old girl born to a G1P1 mother. After delivery, she was found to have a hypoglycemic episode with no obvious history of maternal diabetes, a hemoglobin level of 3.7 g/dL, PLT (platelets) of 32,000/UL, and a positive Coombs test. As a result of mistaking the symptoms for autoimmune hemolytic anemia, a transfusion of RBC and platelet concentration was performed with intravenous (IV) and oral administration of prednisolone. Retesting of laboratory blood work revealed improvement in HB levels to 7.5 g/dL and PLT count to 89,000/UL. Her physical examination was normal, blood smear showed hypochromatic RBC, poikilocytosis, and elevated levels of WBC. However, it was ruled irrelevant because it was carried out after RBC and PLT transfusion. During hospitalization at that time, she was tested for CMV, EBV, and HBV, all of which turned out to be negative. A sudden hyperglycemic episode (blood sugar level of 354 mg/L) was noticed during IV administration of methylprednisolone. It was interpreted as a side effect of steroid use and was treated with fast‐acting insulin subcutaneous injections.

At three and half months of age, she visited a private clinic due to the abrupt onset of pallor, purpura, and bruises on all extremities. She was diagnosed with autoimmune hemolytic anemia and immune thrombocytopenia purpura and was given 1 mg/kg of oral prednisolone with gradual tapering over two‐month period. She did not return to the hospital after this time. She, unfortunately, passed away at the age of one and a half owing to a ketoacidosis‐induced coma.

## 4 CASE 2

The sibling of the first case is a three‐month‐old female infant, who visited the hospital. She was delivered at full‐term by elective cesarean delivery. The delivery went smoothly. Her psychomotor development is normal. She presented to the hospital due to pallor, and dehydration following an upper airway infection. On admission test, results showed hyperglycemia, 270 mg/L;HBA1c of 8.2%; RBCs, /mm^3^; WBCs, /mm^3^ with lymphocyte predomination; PLT, mm^3^; and HB of 5.3 g/dL. Physical examination showed a 7‐kg infant with a height of 63 cm and a head circumference of 42 cm.

**FIGURE 1:**
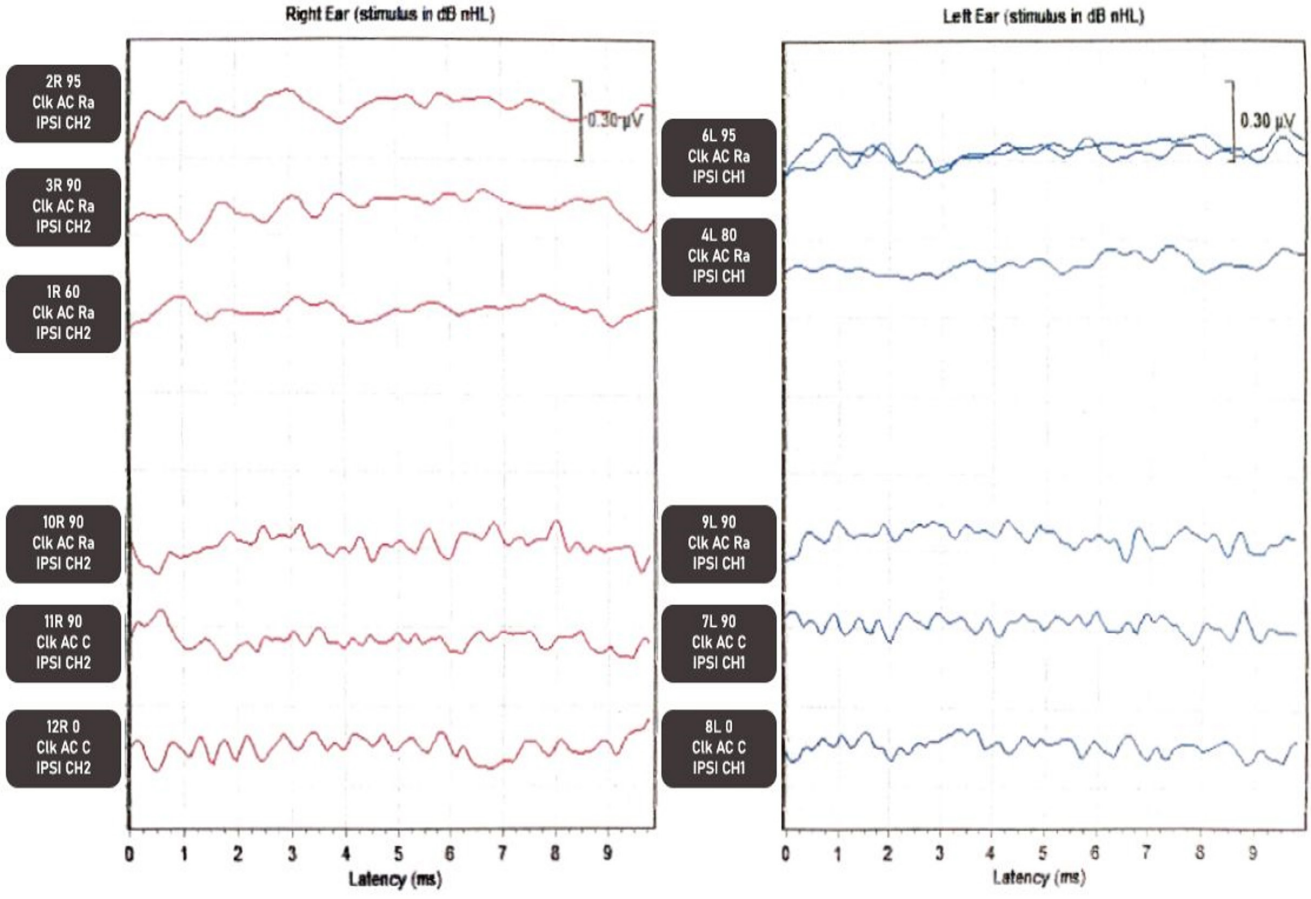
Auditory brainstem response test shows bilateral sensorineural hearing loss for the second child.

Fluid resuscitation, RBC transfusion, and rapid‐acting insulin subcutaneous injection were used to stabilize her. There were no noteworthy findings on abdominal ultrasound and echocardiography. A peripheral blood smear revealed hypochromatic RBC with anisocytosis, poikilocytosis, histiocytes, and targets cells. The cellularity of the bone marrow aspirate was normal with enhanced megaloblastic erythroblasts. Ferritin levels were low in the iron profile. TSH level was 1.2mLU/L, C peptide was 1.1 ng/mL, VIT B9 was 20 ng/mL, and VIT B12 was 365 pg/mL, all within the normal limits. Additional LFT and BUN/Cr tests revealed no abnormalities. The auditory brainstem response test shows a bilateral sensorineural hearing loss (Figure 1).

She was started on thiamine 100 mg t.i.d and rapid‐acting subcutaneous insulin injections of 1.5 units 3 t.i.d. Once a TRMA diagnosis was made. As result, her symptoms considerably improved. She was also referred to an ENT specialist for a hearing aid fitting and an ophthalmologist for fundoscopy evaluation. Due to a lack of resources, no genetic testing was carried out. However, the presence of TRMA symptoms and an improvement in thiamine therapy were enough to confirm the diagnosis. The history of identical symptoms in the deceased sister that we discussed above further strengthens the diagnosis, which we believe that she was misdiagnosed and died as a result of a lack of adequate treatment.

## 5 DISCUSSION

Thiamine pyrophosphate acts as a cofactor for various enzymes involved in oxidative decarboxylation reactions. Mitochondrial pyruvate dehydrogenase, alpha‐ketoglutarate dehydrogenase complexes, and branched‐chain ketoacid dehydrogenase are some of these enzymes.^2^ Thiamine transporters facilitate the transportation of thiamine in a variety of human tissue including pancreatic tissue, bone marrow, brain cells, heart, retina, and skeletal muscles.^3^ Beriberi disease and Wernicke–korsakoff syndrome are both examples of thiamine deficiency‐acquired diseases. However, several inborn errors of metabolic abnormalities involving thiamine transport and other pathways have been reported in the literature. The *SLC19A2* gene encodes thiamine transporter protein. The clinical symptoms of TRMA are caused by a mutation in the *SLC19A2* gene, which is found on the long arm of chromosome 1q23.3. Mutation results in dysfunctional shorter thiamine transporter that disrupts the transportation of thiamine into the cell.^4^ Thiamine‐responsive megaloblastic anemia is an autosomal recessive disease that occurs more frequently in consanguineous marriages. The disease was first described by Roger et al in 1969. However, the pathophysiology of the disease is not fully understood; nevertheless, it has been suggested that some cells have alternative methods to facilitate the entry of thiamine except for erythropoietic cells, insulin‐producing cells, and cells of the inner ear,^4^ which leads to the presence of gene mutation to thiamine paucity in those cells and a cascade of its complications.

Thiamine‐responsive megaloblastic anemia is characterized by megaloblastic anemia, nontype 1 diabetes, and sensorineural hearing loss. The onset of symptoms can span from infancy to adolescence,^1^ and cofactor depletion varies in speed between different tissues that explains why different tissues are affected in different ways.^2^ The megaloblastic anemia can be explained by the role of thiamine in DNA metabolism and heme synthesis in which interruption of the DNA synthesis can lead to large cell anemia,^6^ due to this disruption in some cases thrombocytopenia was noticed; however, leucopenia was rarely noticed due to different needs of hematopoietic progenitor cells to the intracellular thiamine.^6^ In our case, the second patient had hypochromatic anemia due to co‐presenting iron deficiency anemia that masked the megaloblastic anemia. However, bone marrow aspiration showed megaloblastic erythrocyte progenitor cells. In most cases, bone marrow aspiration shows megaloblastic cells and sideroblast, sideroblasts were not found in the second patient, a pharmacological dose of thiamine (50–100 mg) can correct the megaloblastic anemia, but the macrocytic cells persist.^1^ According to the literature, there are two types of transportation systems related to thiamine, when food only sources the thiamine concentration in the intestinal lumen is low; therefore, the activated system is a high‐affinity transporter that requires energy, and it was found to be defective in TRMA syndrome. The second system is activated when the thiamine is available at a high concentration (>2 mmol/L), and the second system is triggered, which involves passive thiamine diffusion.^2^ Thiamine‐responsive megaloblastic anemia patients lack the active absorption mechanism, but, the passive component is present, resulting in a cascade of thiamine deficient consequences when food is the only supply of thiamine. However, absorption can occur in the presence of a high concentration of thiamine.^2^ This method best explains the improvement of the TRMA patient when given higher than dietary doses of thiamine.

Lack of thiamine in insulin‐producing cells can lead to hyperglycemia (nontype 1 diabetes), and thiamine is required by pancreatic B‐cells for glucose transport across the cell membrane, physiological consumption, and insulin secretion.^5^ Oral hypoglycemia medications can help patients with diabetes in its early stages, although most people eventually become insulin‐dependent.^1^ Although initiating thiamine therapy can lower the need for insulin injection and in the majority of cases can diminish the need for it completely, some patients still require insulin treatment during puberty.^6^ In our case, both sisters developed hyperglycemia, which resulted in ketoacidosis and necessitated the use of subcutaneous insulin therapy. The progression of diabetic ketoacidosis is accelerated by misdiagnosis and late commencement of thiamine therapy; therefore, early diagnosis and treatment are beneficial. In our first case, the patient presented with hypoglycemia at delivery with no known history of diabetes nor gestational diabetes in the mother, we could not determine whether there was a link between hypoglycemia after delivery and thiamine transporter mutation. The symptoms of this patient were initially misdiagnosed as autoimmune hemolytic anemia, but treatment for autoimmune hemolytic anemia did not relieve her symptoms. What is more devastating is the subsequent misdiagnosis at an older age due to too much reliance on the previous diagnosis, which resulted in deterioration of symptoms and death. Misdiagnosis of diseases is a major contributor to framing bias, which can lead to unpleasant outcomes in some situations, such as our first scenario.

Another prominent clinical attribute is sensorineural deafness, which does not improve on thiamine therapy, even though some researchers claim that starting thiamine early in infancy can prevent the development of deafness.^6^ Its precise commencement is yet to be determined as it often precedes the diagnosis of TRMA. The hearing loss is irreversible due to disrupted development of the inner ear parts due to thiamine deficiency during infant development. The suitable treatment is the implantation of hearing devices,^4^ and other manifestation of TRMA has been documented in the literature, such as optic atrophy, con‐rod, and retinal dystrophy, which are commonly found in older children with late diagnosis of TRMA. Optic findings were not found in our two cases; however, vigilant observation and regular inspection of ophthalmologic symptoms are important. Cardiac anomalies were also explained by TRMA diseases such as ASD.^2^

## 6 CONCLUSION

Although consanguineous marriage is prevalent in Syria, our two instances are the first to be documented; we believe this is due to a lack of awareness of the condition and its rarity. Thiamine‐responsive megaloblastic anemia is frequently misdiagnosed or goes undetected. Unfortunately, our first case was misdiagnosed and treated incorrectly, resulting in illness progression and death. Misdiagnosis of these diseases primarily contributed to framing bias, such as the case in our first scenario. We emphasize the necessity of developing diagnostic criteria that include all possible differential diagnoses without relying too heavily on what has already been identified; by using this strategy, many incorrectly diagnosed disorders can be discovered and treated correctly.

## AUTHOR CONTRIBUTIONS

**Yara Deeb:** Conceptualization; writing – original draft; writing – review and editing. **Nafiza Martini:** Conceptualization; data curation; writing – original draft; writing – review and editing. **Yara Ahmad Ahmad:** Conceptualization; data curation; writing – original draft; writing – review and editing. **Marwa Alkosti:** Data curation; writing – review and editing. **Othman Hamdan:** Data curation; writing – review and editing.

## CONFLICT OF INTEREST STATEMENT

No conflict of interest.

## ETHICS STATEMENT

Not applicable.

## CONSENT FOR PUBLICATION

Written informed consent was obtained from the parents of the child for publication of this case report and any accompanying images and videos. A copy of the written consent is available for review by the editor of this journal.

## Data Availability

Not applicable
